# 

**Published:** 1807-03

**Authors:** 


### NEW MEDICAL PUBLICATIONS.

The arguments in favour of an Inflammatory Diathesis in Hydrophobia considered; with Selections on the Nature and Treatment of the Disease. By Richard Pearson, M. D. 1s. 6d.

Selections from the Gentleman's Magazine, which commenced in 1731, to the present Time, whence may be deduced the various Cases of Hydrophobia. 2s.

Cautions and Reflections on Canine Madness; with the Method of preventing the Hydrophobia in Persons who have been bitten. By George Lipscombe, Surgeon. 1s.

---

### To CORRESPONDENTS.

The Continuation of the Rev. Mr. Hill's paper, on Vaccination, was received too late for this Month's publication, but it will appear in our next Number.

Communications are received from Mr. Earnest, Mr. Hargreaves, Dr. Kinglake, Crito, Medicus, and Philo-Veritas.

